# Bilateral Ureteral-Iliac Artery Fistula in a Patient with Chronic Indwelling Ureteral Stents: A Case Report and Review

**DOI:** 10.1155/2015/826760

**Published:** 2015-05-05

**Authors:** Arash Rafiei, Timothy A. Weber, Michael Kongnyuy, Raul Ordorica

**Affiliations:** ^1^Tampa General Hospital, Tampa, FL 33606, USA; ^2^University of South Florida Morsani College of Medicine, Tampa, FL 33612, USA

## Abstract

Ureteral-arterial fistula (UAF) is an exceedingly rare but life-threatening condition warranting emergent intervention. Prompt recognition and management of UAF in suspect patients presenting with gross hematuria are required for a successful outcome. We report a rare subset of UAF involving the bilateral common iliac arteries. The patient underwent successful endovascular stent-grafting to correct the arterial defect and delayed open repair of the ureteral strictures. Timely management has benefited from the collaboration of the involved medical teams, which included emergency medicine, urology, and interventional radiology.

## 1. Introduction

Ureteral-arterial fistula (UAF) is a rare but life-threatening condition requiring emergent treatment. Those conditions that either promote inflammation or lead to the breakdown in the integrity of the ureteral or vascular wall can contribute to UAF development. We report our experience with a patient who had received extensive pelvic radiation who then required chronic bilateral ureteral stenting following ileal conduit urinary diversion. It is this setting where our patient developed simultaneous presentation of bilateral UAF involving the common iliac arteries. This patient was successfully managed with initial endovascular stent-grafting followed by open repair of bilateral ureteral stenosis.

## 2. Case History

**Figure 1:**
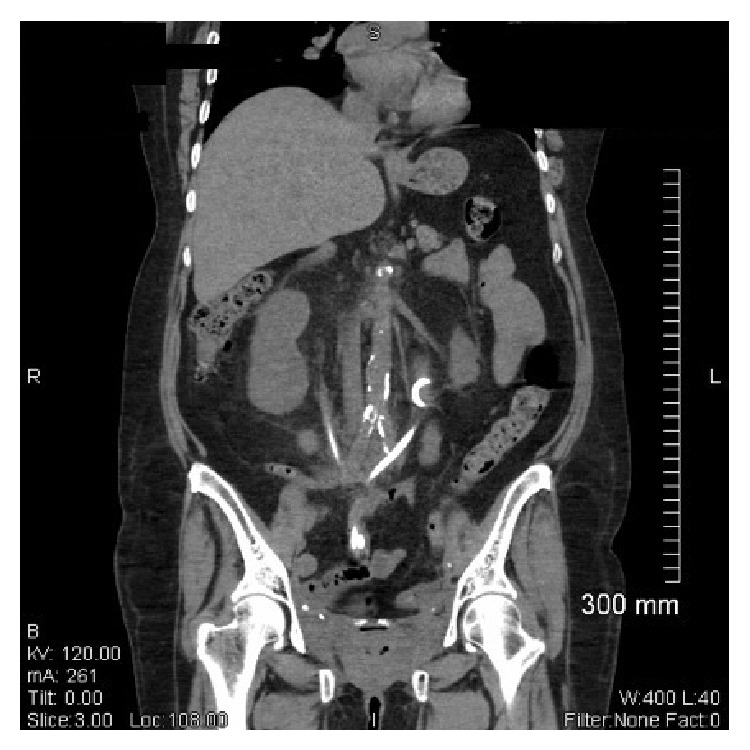
Sagittal view of patient's CT scan, without contrast, showing mild bilateral hydronephrosis and proper stent placement through the conduit with no evidence of pseudoaneurysm of the distal aorta or iliac vessels.

A 58-year-old woman with a history of refractory radiation cystitis after radiation treatment for cervical cancer underwent simple cystectomy, ileal conduit urinary diversion (Wallace anastomosis), and bilateral 7 F JJ ureteral stents in 9/2009. Two months following her surgery, the patient was found to have bilateral ureterointestinal anastomotic strictures requiring antegrade ureteral stent placement; these were 10 F biliary drain stents placed by interventional radiology. At three months she underwent scheduled retrograde stent placement with 10 F JJ stents, 10 F JJ because they were replacing the 10 F biliary drain stents. At two weeks following this most recent stent exchange, the patient developed left flank discomfort, gross hematuria for which she presented to the emergency department. The patient was not taking any anticoagulants. Physical examination revealed some clots within the conduit with some apparent trauma caused by the stents at the level of the stoma. Her hemoglobin was stable at 9.5 and CT scan revealed mild bilateral hydronephrosis and proper stent placement through the conduit with no evidence of pseudoaneurysm of the distal aorta or iliac vessels (Figure 1). Following discharge from the emergency department she represented within 24 hours with identical complaints. Physical examination revealed a heart rate of 114 and a blood pressure of 83/54. Clot was once again observed in the conduit; however, her hemoglobin had dropped from 9.5 to 6.3. Her serum creatinine was 1.3 and her coagulation profile was normal. The patient was immediately brought to the operating room for a looposcopy and bilateral retrograde pyelography to determine the source of the hematuria. After passage of a guide wire through the left side and removal of the stent, brisk bleeding developed from the conduit resulting in a precipitous drop in blood pressure requiring immediate resuscitation. A Fogarty balloon was left in the left midureter, inflated to 1 mL, and pressure was maintained over the stoma of the conduit and the patient was transported to the interventional radiology suite for possible embolization.

**Figure 2:**
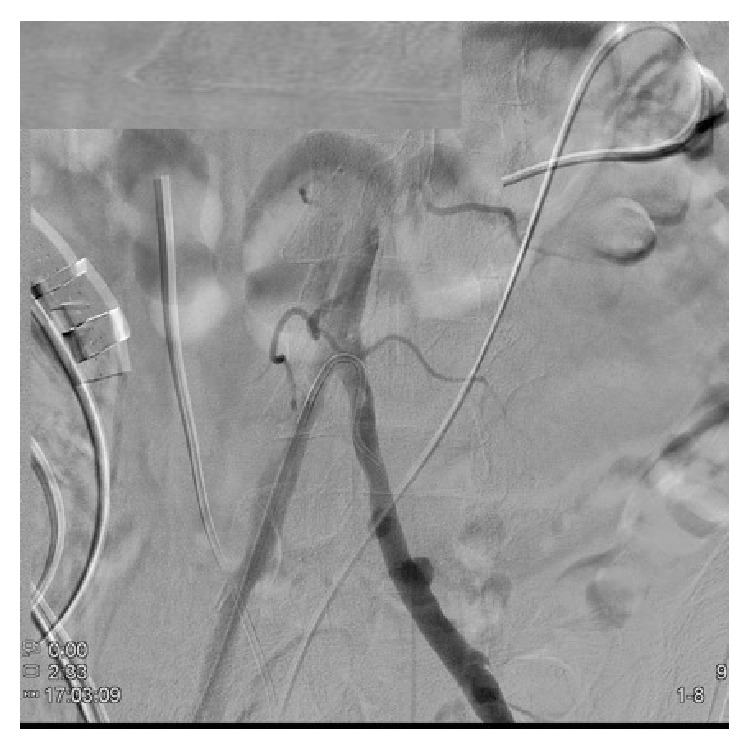
Patient angiogram demonstrating multiple areas of ruptured plaque and extravasation on the left common iliac artery.

**Figure 3:**
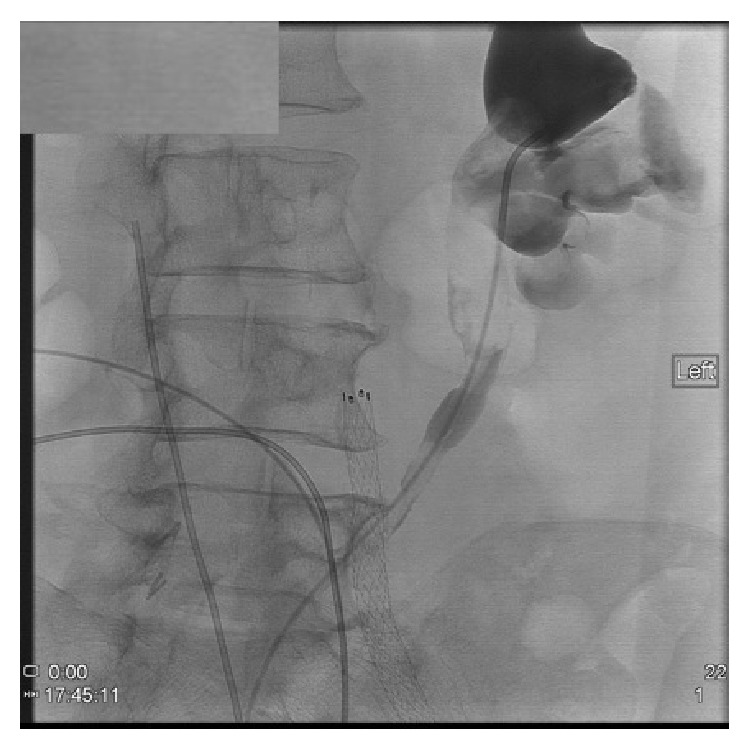
Angiography showing an endovascular stent placed in the left common iliac artery.

In the interventional radiology suite, the patient underwent bilateral provocative angiography of the left and right common iliac arteries using the SOS Omni catheter. On the left, contrast injection demonstrated multiple areas of ruptured plaque and extravasation (Figure 2). An 8 mm × 80 mm FLUENCY stent graft was advanced and deployed into the left common iliac artery just above the hypogastric artery take-off and the stent was dilated to 7 mm (Figure 3). At this point, the left ureteral stent was exchanged without mishap. However, when attempting to change the right ureteral stent, blood began to extravasate from the conduit again. A catheter was quickly placed into the right common iliac artery and injection of contrast demonstrated another ulcerated plaque at the level of the right ureter. Another 8 mm × 80 mm FLUENCY stent graft was deployed and angioplastied to 7 mm. Further interrogation showed patent arteries without any further ulceration.

Approximately four months after the endovascular stent placements a MAG 3 renal scan showed reduced left renal function (14%). Given the continued presence of the bilateral ureteral stents and the newly added vascular stents, there was concern for further erosion and fistula formation. The patient subsequently underwent open correction at which time the bilateral ureters were found to be densely adhered to the common iliac arteries. Successful dissection of each ureter was completed enabling left nephroureterectomy and resection of the right distal strictured ureter with revision of the ureteral-intestinal anastomosis. The right ureter has, after revision, remained patent with no strictures. She has been healthy since that time and has not required any further urological interventions.

## 3. Discussion

Ureteral-arterial fistula (UAF), however rare, carries the risk of death secondary to exsanguination. The true incidence of UAF is unknown, but there are increasing reports in the literature. Less than 20 cases were reported prior to 1994, and since then case reports have doubled. Patient presentation with bilateral UAF is quite rare, with the first report in Germany in 1994 [1].

The most important risk factors for development of UAF include previous pelvic surgery, radiation therapy, and chronic ureteral stents [2]. Other risk factors include coronary artery disease, vascular disease, infection, and pregnancy [3–5].

The pathophysiology of UAF formation is theorized to be secondary to disruption of the vasa vasorum leading to inflammation and subsequent fibrosis. Pressure necrosis of the ureter occurs and a pulsatile arterial pressure is counterbalanced against a stented ureter [6]. The pressure head of arterial pulsion on a weakened arterial wall against a scarred and fibrotic stented ureter may facilitate fistula formation [7]. Physical stimulation and disruption during stent exchange precipitate fistula enlargement. To our knowledge, no correlation has been made between radiation dosage and UAF with indwelling stents, although one report found greater arterial injury with increasing dosages of radiation [8].

Fistulas can present anywhere along major branch chain of the distal system with reports of fistula formation along common iliac artery (37%), external iliac artery (37%), and internal iliac artery (25%) [6]. Prior to development of the J-J in 1978, few cases of UAF were reported, suggesting that a combination of increased stent usage and radiation therapy is responsible for this unfortunate outcome [9].

A high index of suspicion is necessary for early diagnosis and disaster prevention. One study reported up to 52% mortality in undiagnosed cases of UAF [10]. Additionally a multidisciplinary approach with good communication between the surgeon and the interventionalist is required in dealing with this rare complication. Diagnosis traditionally included retrograde pyelogram and ureteroscopy, but these have limited value in the acute setting and may worsen the situation. CT has a low sensitivity for this complication, and the radiologist must be keen on the presence of pseudoaneurysms, which may be difficult to assess because of interference from high-density ureteral catheters. If no extravasation can be seen on CT, then standard angiography is diagnostic in 23%–41% of cases [5, 11]. Provocative angiography is the most sensitive technique to assess extravasation and involves removal of the indwelling stent during angiography. However, one must maintain suspicion if a provocative angiogram is negative in the face of persistent hematuria.

Multiple treatment options exist and management should be tailored to the patient's clinical status. Approaches have included open vascular repair, surgical bypass grafting, embolization, ligation, and endovascular stenting with an increase in employment of interventional modalities including endovascular stent grafts which was first introduced in 1996 by Kerns et al. [12, 13]. IR methods included embolization, embolization plus surgical bypass grafting, and endovascular repair with stent grafts. Although minimally invasive endovascular treatment is gaining popularity and short-term results are promising, there is real concern for stent fracture and infection [7]. Removal of offending factors such as indwelling ureteral stents should help to safeguard the vascular prosthetics.

The presence of bilateral UAF heightens the concern for hemodynamic changes and mortality. Our case report highlights the need for timely diagnosis and resuscitation. This patient was successfully treated due to the close collaboration between urology and interventional radiology to allow for seamless management. Also, a 10 F biliary drainage stent could have enabled the formation of the fistula. While the ureteral defect continued to be successfully managed with stenting, appropriate concern was given for the continued risk of erosion. Therefore, long-term management required removal of the ureteral stents with performance of either long-term nephrostomy tube drainage or open repair. Faced with these options, the patient chose open repair regarding which function of the left renal unit was not believed to be sufficient to warrant salvage.

## 4. Conclusions

Given the increasing use of chronic indwelling ureteral stents coupled with radiation treatment, there is increasing evidence of UAF. The high death rate is primarily related to a delay in diagnosis and treatment. Currently, there is no published diagnostic management of UAF based on large prospective studies. However, awareness of this condition as well as knowledge of proper diagnostic modalities will aid in patient management and reduce mortality.

## Abbreviations

UAF: Ureteral-arterial fistula
CT: Cat scan
IR: Interventional radiology.
